# The Maxillomandibular Sagittal Assessment: The ABwise Appraisal and Its Correlation with ANB Angle

**DOI:** 10.3390/jcm14041379

**Published:** 2025-02-19

**Authors:** Elisa Boccalari, Ornella Rossi, Benedetta Baldini, Cinzia Tripicchio, Marco Serafin, Alberto Caprioglio

**Affiliations:** 1 Department of Biomedical, Surgical and Dental Sciences, University of Milan, Via della Commenda 10, 20122 Milan, Italy; elisa.boccalari@unimi.it (E.B.); cinzia.tripicchio@unimi.it (C.T.); alberto.caprioglio@unimi.it (A.C.); 2 Department of Electronics, Information and Bioengineering, Politecnico di Milano, 20122 Milan, Italy; benedetta.baldini@polimi.it; 3 Department of Biomedical Sciences for Health, University of Milan, 20122 Milan, Italy; marco.serafin@unimi.it; 4 Fondazione IRCCS Cà Granda, Ospedale Maggiore Policlinico, 20122 Milan, Italy

**Keywords:** maxillomandibular sagittal assessment, ABwise measurement, ANB angle correlation, three-dimensional cephalometry, CBCT in orthodontics, skeletal class classification

## Abstract

The ANB angle, the cephalometric parameter of choice for assessing the anteroposterior relationship between the maxilla and mandible, is subject to several limitations, prompting the investigation of alternative parameters. **Objective:** This study aimed to investigate the ABwise measurement as an alternative to the ANB angle for evaluating maxillomandibular relationships in orthodontics, particularly addressing the impact of skeletal discrepancies on conventional methods. **Methods**: A retrospective analysis was performed on a CBCT dataset of patients attending the University of Milan’s Department of Orthodontics and Maxillofacial Surgery, selected based on high-quality imaging, a full-cranium field of view, and a slice thickness between 150 and 300 μm. Eight craniofacial landmarks were annotated using the 3D Slicer software to calculate the ANB values and the new ABwise measurement. Statistical analyses included Spearman’s correlation (ρ), linear regression, and inter-rater agreement (Cohen’s κ score), with data classified into skeletal Classes I, II, and III based on defined thresholds. **Results:** 354 CBCT were selected and analyzed (mean age: 18.6 years). ABwise showed a strong correlation with the ANB angle (ρ = 0.805) and new normative ranges for ABwise were established: Class I (−1.4 ± 2.3 mm), Class II (>0.9 mm), and Class III (<−3.7 mm). Moderate agreement was observed between the ABwise and ANB classifications (κ = 0.527). ABwise effectively addressed limitations associated with divergence and vertical discrepancies, providing a more reliable assessment of skeletal sagittal relationships. **Conclusions:** ABwise presents a viable alternative to the ANB angle for three-dimensional cephalometric analysis, offering improved accuracy and alignment with radioprotection principles by reducing the CBCT field of view needed for its measurement. Further research is required in order to validate these findings across diverse populations and clinical scenarios.

## 1. Introduction

Diagnosis in orthodontics is the key for planning a correct therapeutic treatment plan. Radiographic exams are essential to the performance of orthodontic treatments through the creation of a cephalometric analysis, a quantitative diagnostic tool that is used by orthodontists to evaluate skeletal and dentoalveolar relationships, morphometrical characteristics, and the growth patterns of patients [1]. Cephalometric tracings on lateral radiographs have been the diagnostic method of choice in orthodontics since their appearance in the 1930s [2,3].

In cephalometric analysis, the ANB angle, which measures the anteroposterior relationship between the maxilla and mandible regarding the anterior cranial base, is subject to several significant limitations [4,5,6,7,8,9,10]. Individual variability, including differences in growth patterns that influence vertical facial height, can significantly impact both the accuracy and interpretation of the ANB angle, underscoring the need for alternative assessment methods. For instance, vertical patterns can lead to misleading ANB values, and normative values may differ across ethnic groups, complicating its clinical application. Skeletal discrepancies may alter the angle without reflecting true anteroposterior discrepancies [10]. In fact, divergency, referring to the vertical skeletal relationship between the cranial base and the mandibular plane, can significantly impact the ANB angle; hyperdivergent and hypodivergent growth patterns can respectively increase or decrease the ANB angle, potentially masking true skeletal relationships [11]. Additionally, skeletal bite classifications, such as deep bites or open bites, can also alter the relative positions of the jaws and subsequently affect the ANB measurement. Moreover, the reliability of the ANB angle is compromised by challenges in accurately identifying anatomical landmarks on cephalometric radiographs, resulting in variability and reduced reproducibility of measurements [9,10,11,12]. The interpretation of the ANB angle is complex, and it must be integrated with other cephalometric parameters, such as the Wits appraisal, to provide a comprehensive assessment of skeletal relationships [7,8]. Despite the well-known limitations of the ANB angle, there is a lack of alternative parameters that are both validated and widely accepted in the literature. Also, the Wits appraisal and its modifications suffer the same misclassification when skeletal or dental compensation exists. Several methods have been proposed to overcome these limitations [5,13,14].

Cone-Beam Computed Tomography (CBCT) significantly enhances cephalometric analysis and the accuracy of ANB measurements by providing three-dimensional imaging of craniofacial structures. Unlike traditional two-dimensional cephalometry, CBCT offers a detailed and precise visualization of skeletal anatomy. Consequently, CBCT helps overcome the limitations of conventional ANB measurements, offering a more comprehensive and reliable evaluation of the anteroposterior jaw relationship [14]. While CBCT offers significant advantages in medical imaging, it also presents challenges related to radioprotection. The selection of a field of view (FOV) is crucial; while a full-cranium FOV may capture more anatomical detail and landmarks, it also exposes patients to higher radiation. This issue raises concerns about radioprotection, particularly in young patients. Therefore, it is essential to balance the need for 3D cephalometry with the principle of radioprotection, beginning with the optimization. Clinicians must carefully select the appropriate FOV, reducing it to an ultra-reduced one limited to the orthodontic region of interest, which ensures adequate diagnostic information while possibly avoiding extra maxillary landmarks typically used to calculate the skeletal class [2,5,15,16,17].

The purpose of this research was to explore new methods of assessing maxillomandibular relationships, especially with a procedure that addresses the interference of divergence. Therefore, the aim was to correlate and validate the use of a new measurement, ABwise, comparing it to the conventional ANB angle using a CBCT dataset. In addition, the secondary outcome was to present an alternative approach for 3D cephalometric analysis based on radiation protection principles.

## 2. Materials and Methods

### 2.1. Ethical Considerations

The Ethical Committee of the University of Milan approved the retrospective data collection (protocol number 71/22, dated 21 July 2022), ensuring compliance with the World Medical Organization’s Declaration of Helsinki principles. All participants provided informed consent for the scientific use of their data prior to undergoing radiological examination.

### 2.2. Participants and Data Collection

The present validation study evaluated a CBCT dataset of 371 scans from patients who underwent orthodontic and maxillofacial treatment from September 2017 to July 2023 at the University of Milan’s Department of Orthodontics and Maxillofacial Surgery. The CBCTs were retrospectively chosen according to specific inclusion criteria: high-quality images, a complete cranium field of view encompassing all required landmarks, and a slice thickness between 150 and 300 μm. Patients exhibiting severe asymmetry, those with a history of orthognathic surgery, or those with systemic diseases or syndromes were dropped from the study. DICOM files from each patient were imported and analyzed using specialized software (3DSlicer, Brigham and Women’s Hospital, Boston, MA, USA, version 5.2.2) for the manual annotation of eight landmarks (seven unilateral and one bilateral) [18,19].

Each landmark was identified on 2D axial views (x-axis, y-axis, and z-axis) to ensure precise localization. Subsequently, each point was verified on the 3D rendering, which allowed visual confirmation of its position. For accurate landmark identification, standardized criteria were applied for each reference point. For example, the Gonion and Menton points were located based on the mandibular curvature, while the ANS point was identified as the most anterior point of the anterior nasal spine, and the PNS as the most posterior point of the posterior nasal spine. Detailed information about the landmarks and their definitions is provided in Table 1.

### 2.3. Statistical Analysis

After manually annotating the landmarks, the coordinates of each reference point were exported and processed using programming software (Python, version 3.11.4). Subsequently, the ANB and ABwise measurements were computed. The ANB angle was calculated using the following formula:

ANB = SNA − SNB

**Figure 1:**
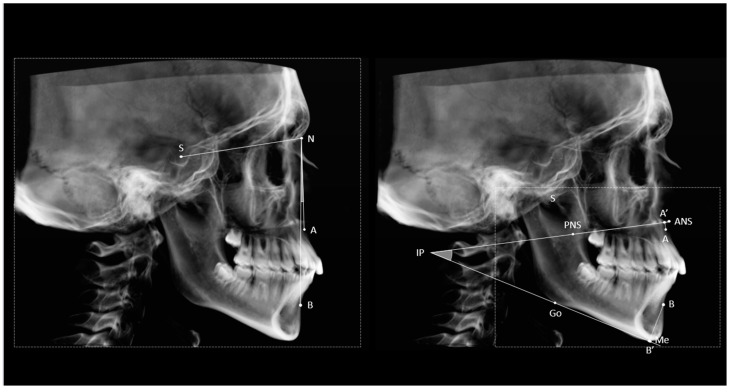
Cephalometric template representing the construction between ANB (**left**) and ABwise (**right**) measurements. Transparent areas underline the difference in the required FOVs of the two techniques.

The ABwise measurement involved a preliminary step: first, a mandibular plane (MP) was established, passing through the right and left Gonion and Menton landmarks. Next, the intersection point between the MP and the bispinal line (BL), which passes through ANS-PNS, was identified as the intersection point (IP). Figure 1 shows a graphic representation of the construction methods for ANB and ABwise. The projections of points A and B onto the BL line (A’) and the mandibular plane (B’) were then constructed. Finally, the distances from A’ and B’ to the IP were used to calculate the ABwise by the following formula:

$$ABwise=\left(A^{\prime }-intersection\;point\right)-(B^{\prime }-interesection\;point)$$

To determine the appropriate sample size for correlation analysis, a power calculation was performed based on data from a pilot study of 50 cases, which yielded a correlation coefficient of 0.687. The Fisher Z-transformation method was used to convert the correlation into an effect size. A two-tailed test was conducted with a significance level of 1% (α = 0.01) and a statistical power of 99% (β = 0.01) to minimize the risk of Type I and Type II errors, respectively. The analysis determined that a minimum of 68 cases was required to achieve sufficient statistical power, ensuring the reliability of the correlation findings.

To calculate the method error and intra-observer reliability for the tested variables, Dahlberg’s method was used. The variability was quantified with values ranging from 0.2° to 1.2° for angular measurements and from 0.2 mm to 0.8 mm for linear measurements. Based on these results, a low degree of variability was observed, deeming the measurements reliable for further analyses.

First, the normality of the data was tested using the Shapiro–Wilk test to determine whether to use parametric or non-parametric correlation tests. Since the two samples showed significant *p*-values (*p* < 0.001), the data were considered non-normally distributed and non-parametric tests were therefore chosen. Additionally, outlier cases were identified and excluded using the Tukey’s fences method, based on the Interquartile Range (IQR) by a factor of 1.5.

Inferential statistical analysis included the Spearman’s correlation coefficient (*ρ*) to assess the strength and direction of the relationship between the angular measurement ANB (independent variable) and the proportional measurement ABwise (dependent variable). Statistical significance was considered at *p* < 0.05, and coefficients *ρ* > 0.70 were deemed strong and clinically relevant. Linear regression was used to test the relationships between the variables, providing the transforming equation between them.

Finally, considering the transformation equation and the subsequent threshold for the ANB angle, the data were classified into Classes I, II, and III. Therefore, the inter-rater agreement between the two methods was evaluated by contingency table and Cohen’s kappa score (κ).

## 3. Results

Outlier detection highlighted 17 CBCTs, which were removed from the dataset to standardize the statistical calculations. Therefore, the correlation among the ANB angle and the ABwise was tested on 354 patients (212M, 142F. Mean age: 18.6 ± 8.8 yrs. Range: 6.4–48.2 yrs), consecutively selected. The sample was characterized by a mean ANB value of 3.82 ± 2.78° (range: −6.46–12.56°) and a mean ABwise value of 0.68 ± 4.29 mm (range −18.24–13.22 mm).

To provide the new clinical norms ranges for the measurements obtained by ABwise, the strength and direction of the relationship were first evaluated using a non-parametric Spearman correlation coefficient. The analysis showed a high correlation (ρ = 0.805) and was statistically significant (*p* < 0.001); thus it was considered clinically relevant. Post-hoc analysis by a Siegel estimator for non-parametric linear regression was used to model the relationship between the two sets of measurements. The obtained equation was the following:

ABwise=1.17×ANB−3.78

**Figure 2:**
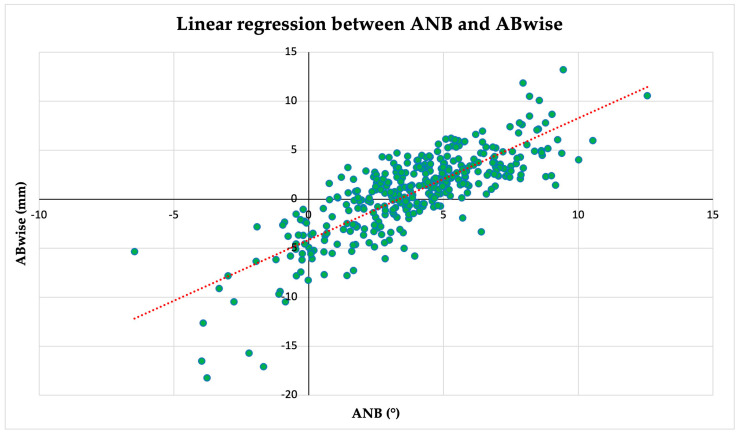
Siegel linear regression graph between independent (**x**, ANB) and dependent (**y**, ABwise) variables. The model reveals a strong correlation (ρ = 0.805, *p* < 0.001) and explains 64.9% of the variance (R^2^ = 0.649). The regression equation is ABwise = 1.17 × ANB—3.78.

Therefore, the rounded corresponding norm of ABwise to ANB (2 ± 2°) assessment was −1.4 ± 2.3 mm to classify the sagittal discrepancy as Class I; values lower than −3.7 mm correspond to a Class III relationship, whereas values higher than 0.9 mm correspond to a Class II relationship. Figure 2 represents the linear regression between the included parameters.

The analysis revealed a moderate level of agreement between the ANB and ABwise classifications, as indicated by a κ score of 0.527. The agreement is substantial enough to suggest some consistency between the two methods, but it also indicates that there are some differences in how they classify cases. Additionally, the correlation analysis between the ANB and ABwise yielded an R^2^ value of 0.649, suggesting that approximately 64.9% of the variance in one metric can be explained by the other.

Finally, contingency table (Table 2) highlighted the best concordance in Class II cases, followed by Class I cases. Notably, there were no misclassifications between Class II and Class III cases, whereas the other misclassifications contributed to the moderate κ score.

The classification performance of ABwise compared to ANB revealed an overall accuracy of 73%, indicating a strong but not perfect alignment between the two methods. Precision analysis demonstrated that ABwise had the highest classification reliability for Class II cases (precision = 79%), followed by Class I (69%) and Class III (51%), highlighting that Class III had the highest misclassification rate. Sensitivity (recall) values further supported these findings, with Class II achieving the highest recall (87%), meaning that most Class II cases classified by ANB were also classified as Class II by ABwise. In contrast, Class I had a recall of 55%, with a portion of cases misclassified as Class II or III, while Class III had a recall of 68%, suggesting that some Class III cases were reclassified as Class I. Table 3 presents the classification performance of the ABwise assessment.

## 4. Discussion

The findings of this study confirm the potential of ABwise as a more accurate and reliable metric for evaluating maxillomandibular relationships, particularly in addressing the limitations of the ANB angle caused by vertical discrepancies and rotational growth patterns.

In 1975, Jacobson showed that the ANB angle does not provide an adequate assessment of skeletal relationships because the growth rotation of the mandible and the anteroposterior position of nasion to point A and point B may affect it [7,8]. Specifically, the factors affecting the ANB angle are: growth rotation of the jaws; vertical growth reflected in the distance between points A and B; vertical growth reflected in the distance between points N and B; the length of the anterior cranial base; and the anteroposterior position of the point nasion [8,9,10,12,20]. Furthermore, the criticism has been based upon the fact that the nasion is not a fixed point and any change in its position would affect ANB [15]. Unlike the ANB angle, which is influenced by nasion positioning and vertical growth patterns, ABwise reduces these biases by integrating the mandibular plane and bispinal line, as evidenced by the high correlation coefficient. This aligns with Jacobson’s critique of ANB and supports the need for metrics less reliant on unstable landmarks.

To define whether the ANB angle is reliable, it is necessary to ascertain the relative anteroposterior position of the jaws to nasion and the rotational relationship of the jaws relative to the anterior cranial base [7].

In many instances, the SNA, SNB, and ANB angles accurately reflect the degree of anteroposterior positioning of the jaws in the relationship of the jaws to each other. However, in some cases, these angles are not representative of the jaws’ relationship or disharmony. Jacobson proposed the application of the “Wits” appraisal and demonstrated its clinical utility [7,8]. The Wits appraisal avoids the use of nasion and reduces the rotational effects of jaw growth, but it uses the occlusal plane, which is a dental parameter, to describe skeletal discrepancies. The occlusal plane can be easily affected by tooth eruption and dental development. However, the Wits appraisal does eliminate the geometric effects of using reference points such as the nasion outside the affected area [10].

Jacobson assumed that this appraisal was closer to the area of the dental and skeletal base relations and less prone to variation when compared to the ANB angle. Nevertheless, the occlusal plane is difficult to measure accurately, particularly in adult dentitions in which third molars are present [8]. This plane is not flat but concave. In more mature dentitions, therefore, the plane maximum intercuspation cannot always be used, since this plane follows a turn. A line joining the mesiobuccally cusp of the upper first molar to a point midway between the overlap of the upper and lower incisors is a satisfactory method of standardizing the occlusal plane measurement in most normal occlusions. However, this method is not suitable for occlusions with a deep curve of Spee and for malocclusions with upper or lower incisors over- or under-erupted. Probably the most suitable and convenient method of standardizing the plane of occlusion is to join the midpoints of overlap of the mesiobuccal cusps of the first molars and the buccal cusps of the first premolars [7].

Lastly, the ANB angle alone has limitations in predicting orthodontic or surgical treatment outcomes, as it does not account for the three-dimensional nature of craniofacial structures.

It has been statistically proven that ABwise not only provides a solution for addressing vertical discrepancies but also enhances diagnostic precision by avoiding reliance on reference points like the nasion, which are prone to variability. Compared to ANB, it might mitigate the influence of vertical divergence and skeletal bite.

So, one of the most compelling aspects of ABwise is its ability to account for the vertical dimension’s interference, a shortcoming of the ANB angle, as we previously discussed.

The vertical dimension, particularly in cases of hyperdivergent or hypodivergent patterns as well as deep bite or open bite patients, can significantly skew ANB measurements, leading to potential misdiagnosis. ABwise is an innovative method because by incorporating the mandibular plane and the bispinal line into its calculation, it offers a more complete and real view of the maxillomandibular relationship. By projecting points A and B onto their corresponding lines and then calculating the respective distances from the intersection point, ABwise provides a measure of the anteroposterior relationship that is minimally affected by vertical discrepancies.

When a change in the skeletal vertical dimension occurs (an increase or decrease in the divergence, therefore in the skeletal vertical dimension) it likewise affects the distances from the projections of the A and B points to the intersection points on the maxillary and mandibular planes. Since those distances change synchronously, we can consider the subsequent measurement not affected and not significantly distorted by the change of divergence.

Finally, the secondary outcome of this work was to present an alternative method for 3D cephalometric analysis based on radiation protection principles. After the introduction of CBCT in dentistry in 1998, which enabled a lower radiation dose than multislice computed tomography, several cephalometric three-dimensional tracings were developed [12]. The advent of 3D imaging technology and software made it possible to visualize, study, and evaluate all three dimensions of the craniofacial structure with 3D analysis [2,16]. In spite of the necessity of ABwise in using three-dimensional diagnostic investigations, the reduction in the field of view required for ABwise measurements aligns with the principles of radioprotection in using CBCT technology [21]. By minimizing radiation exposure without compromising diagnostic accuracy, ABwise could represent a safer alternative, particularly for younger patients, who are more susceptible to the stochastic risks associated with ionizing radiation [22].

Despite the promising results of this study, several limitations must be acknowledged. The moderate agreement between the ABwise and ANB classifications indicates that while ABwise is effective in mitigating the impact of vertical discrepancies, there remain inconsistencies, particularly in Class I and Class III cases. The observed inconsistency in misclassification between skeletal classes arises from the inherent differences in the reference planes and anatomical landmarks used by ANB and ABwise. ANB is influenced by vertical growth patterns and cranial base inclination, which can lead to discrepancies in classification, particularly in borderline cases. In contrast, ABwise minimizes these effects by using the mandibular plane and bispinal line. While a moderate agreement was found between the two methods, ABwise provides a different perspective on skeletal classification by reducing the potential impact of vertical discrepancies. The observed misclassifications primarily occur between Class I and Class III cases, likely due to ANB’s susceptibility to variations in divergence. These findings highlight the need for a comprehensive approach to sagittal assessment, integrating multiple cephalometric parameters for accurate diagnosis.

Additionally, the study relied on a single-center CBCT dataset, which may limit the generalizability of the findings across different populations and clinical settings. Another potential limitation is the dependency on CBCT imaging for ABwise measurements. While CBCT offers superior precision, its accessibility and cost may restrict widespread adoption in routine clinical practice [23,24]. Lastly, the manual annotation of craniofacial landmarks introduces the possibility of operator-dependent bias, despite efforts to ensure reliability through intra-observer agreement testing.

## 5. Conclusions

Based on the previous findings, it can be concluded that ABwise can represent an alternative measure for skeletal sagittal assessment. High and significant correlation coefficients were found between ABwise and ANB angles, suggesting potential uses for ABwise in 3D cephalometric analysis. Class I malocclusion is represented by an ABwise value of −1.4 ± 2.3 mm; values lower or higher than that range correspond to Class III and Class II malocclusion, respectively. Despite the promising results, due to the low agreement between ANB and ABwise skeletal Class classifications, further multicenter studies involving diverse clinical scenarios and populations are essential to fully establish the clinical utility and reliability of ABwise.

## Figures and Tables

**Table 1:** Landmarks’ list and definitions.

Landmark	Abbreviation	Definition
Nasion	N	The most anterior aspect of the frontonasal suture, observed on the median sagittal plane
Sella	S	Three-dimensional center of the sella turcica
A point	A	The most posterior aspect of the concavity between the ANS and the alveolar bone of the upper incisors, observed on the median sagittal plane
B point	B	The most posterior aspect of the concavity between the Pogonion and the alveolar bone of the lower incisors, observed on the median sagittal plane
Anterior Nasal Spine	ANS	The most anterior aspect of the nasal spine, observed on the median sagittal plane
Posterior Nasal Spine	PNS	The most posterior aspect of the nasal spine, observed on the median sagittal plane
Gonion	Go	The most postero-inferior point of the gonial angle, observed on the right and left sides of the mandible
Menton	Me	The lowest aspect of the mandibular symphysis, observed on the median sagittal plane

**Table 2:** Contingency table regarding the classification of Class I, II, and III cases between the ANB and ABwise methods. The rows represent the classifications based on the ANB angle, while the columns represent the classifications based on the ABwise measurement. The values within the table indicate the number of cases that fall into each corresponding classification category.

		ABwise			
		Class I	Class II	Class III	
ANB	Class I	77	41	22	140
	Class II	23	157	0	180
	Class III	11	0	23	34
		111	198	45	354

**Table 3:** Precision, sensitivity (recall), and F1-score performance of ABwise compared to ANB. Precision measures the proportion of correctly identified cases within a predicted class, indicating the accuracy of positive predictions. Sensitivity (recall) reflects the model’s ability to correctly detect actual cases within a class, assessing how well true positives are identified. The F1-score, as the harmonic mean of precision and sensitivity, provides a balanced measure of classification performance, especially in cases of class imbalance.

Metric	Class I	Class II	Class III	Overall
Precision	69%	79%	51%	73%
Sensitivity	55%	87%	68%	-
F1-score	61%	83%	58%	-
Overall accuracy	-	-	-	73%

## Data Availability

In accordance with patient privacy regulations and ethical guidelines, the data supporting the findings of this study are not publicly available. However, they can be accessed upon reasonable request. Researchers seeking to obtain the data should contact the authors, providing a clear and justified purpose for the data request. All requests will be reviewed to ensure compliance with patient confidentiality and data protection policies.
